# Complete mitochondrial genome of the harmful algal bloom species *Pseudo-nitzschia delicatissima* (Bacillariophyceae, Bacillariophyta)

**DOI:** 10.1080/23802359.2021.1959441

**Published:** 2021-08-02

**Authors:** Ziyan He, Yang Chen, Zongmei Cui, Mengjia Zhang, Yongfang Zhao, Feng Liu, Nansheng Chen

**Affiliations:** a CAS Key Laboratory of Marine Ecology and Environmental Sciences, Institute of Oceanology, Chinese Academy of Sciences, Qingdao, China; b Laboratory of Marine Ecology and Environmental Science, Qingdao National Laboratory for Marine Science and Technology, Qingdao, China; c School of Earth and Planetary, University of Chinese Academy of Sciences, Beijing, China; d Center for Ocean Mega-Science, Chinese Academy of Sciences, Qingdao, China; e Jiaozhou Bay National Marine Ecosystem Research Station, Institute of Oceanology, Chinese Academy of Sciences, Qingdao, China; f Department of Molecular Biology and Biochemistry, Simon Fraser University, Burnaby, Canada

**Keywords:** Diatoms, mitochondrial genome, *Pseudo-nitzschia delicatissima*, group II intron

## Abstract

*Pseudo-nitzschia* is an important genus of diatoms with many species capable of inducing harmful algae blooms (HABs) in coastal and oceanic waters, some of which produce the toxin domoic acid (DA), a neurotoxin that causes amnesic shellfish poisoning (ASP). *Pseudo-nitzschia delicatissima* is a cosmopolitan species that can induce HABs and produce DA. Nevertheless, mitochondrial genome of *P. delicatissima* has not been revealed. In this study, we determined the complete mitochondrial genome of *P. delicatissima* for the first time. The circular mitochondrial genome was 42,182 bp in length with GC content of 30.37%. It consisted of 65 genes including 39 protein-coding genes (PCGs), 24 tRNA genes, and two rRNA genes. This mitogenome has a group II intron, located in the cytochrome c oxidase subunit genes (*cox1*), with *orf790* identified inside the intron region. Phylogenetic analysis revealed that *P. delicatissima* was clustered well with *P. multiseries.* This analysis is valuable for studying the evolutionary relationships among *Pseudo-nitzschia* species, and for comparative analysis of *P. delicatissima* strains.

Diatoms are a widespread and diverse group of unicellular eukaryotic algae, and they have significant ecological importance in the carbon and silicate cycles, accounting for approximately 20% of the global photosynthetic carbon fixation (Field et al. 1998). *Pseudo-nitzschia* is a species-rich genus of diatoms, many of which can induce harmful algae blooms (HABs) in coastal and oceanic waters and produce the toxin domoic acid (DA), a neurotoxin causing amnesic shellfish poisoning (ASP) (Bates et al. 2018). The species *Pseudo-nitzschia delicatissima* (Cleve) Heiden 1928 has been identified to exist in many regions including Europe, America, Asia, and Oceania (Bates et al. 2018). Furthermore, *P. delicatissima* has been reported to bloom in Gulf of Naples (Italy), Berowra Creek (Australian), Bay of Seine (France), Bizerte Lagoon (Tunisia), Jeddah (Saudi Arabia), Fujian Province (China), South China Sea, and some strains can produce DA (Orsini et al. 2004; Li 2012; Fernandes et al. 2014; Thorel et al. 2017; Al-Aidaroos et al. 2019; Ajani et al. 2020). At present, morphological features of *P. delicatissima* has been characterized. It has lanceolate-shaped cells with 1.5–2.0 μm in width and 19–76 μm in length, containing two plastids and being capable of forming stepped chains (Lundholm et al. 2006; Ghiron et al. 2008). Nevertheless, mitochondrial genome of *P. delicatissima* has not been revealed. In this study, we determined and analyzed the complete mitochondrial genome of *P. delicatissima*.

The strain CNS00135 was isolated from water samples collected from the Jiaozhou Bay, China (36°02'058″N, 120°22'866″E) in October 2019 onboard the research vessel “Innovation”. It was identified to be *P. delicatissima* based on its morphological features and sequence similarity of known molecular markers including *rbcL*, 18S rDNA, and ITS. Previous studies had found large genetic variation among the strains of *P. delicatissima*, and many new species were separated from *P. delicatissima* group (Amato et al. 2007; Quijano-Scheggia et al. 2009). In this study, ML tree based on ITS showed that CNS00135 belong to the same clade with strain Laesø5, which was the epitype of *P. delicatissima* (Lundholm et al. 2006), thus CNS00135 was identified to be *P. delicatissima* sensu stricto. The voucher samples were deposited at the KLMEES of IOCAS (Nansheng Chen, chenn@qdio.ac.cn) under the voucher number CNS00135.

Total DNA was extracted using the modified CTAB method (Doyle 1987). DNA sample were sequenced using the Illumina NovaSeq 6000 platform (Illumina, San Diego, CA, USA). The mitochondrial genome was *de novo* assembled using the program GetOrganelle (Jin et al. 2020), with SPAdes version 3.10.1 as assembler (Bankevich et al. 2012). Subsequently, the obtained genome sequence was checked by aligning sequencing reads against the mitochondrial genome using the MEM algorithm of BWA v0.7.17 (Li and Durbin 2010). Alignments were visualized using IGV v2.8.12 (Robinson et al. 2011). ORF finder (https://www.ncbi.nlm.nih.gov/orffinder) and MFannot (https://megasun.bch.umontreal.ca/RNAweasel/) were used to annotate the mitochondrial genome of *P. delicatissima*.

The complete circular mitochondrial genome (mtDNA, GenBank accession No: MW436413) of *P. delicatissima* was 42,182 bp in length, which was shorter than that of *P. multiseries* (46,283 bp), the only mitochondrial genome of a *Pseudo-nitzchia* species that has been published (Yuan et al. 2016). The GC content of *P. delicatissima* was 30.37% (T 33.52%, C 15.59%, A 36.11%, and G 14.78%), which was lower than that of *P. multiseries* (31.05%). The mtDNA of *P. delicatissima* consisted of 65 genes including 39 PCGs (including three open reading frames, *orf*s), 24 tRNA genes, and two rRNA genes. Among the 39 PCGs, the start codons 35 genes were ATG, while the start codons of *atp8*, *cob*, *rpl5*, and *orf790* were TTG, TTG, ATT, ATC, respectively. The stop codons of 32 PGGs were the canonical stop codon TAA, while the stop codons of *cox1*, *nad4L*, *nad6*, *nad9*, *tatC* were TAG, respectively, and the stop codons of *rpl5* and *orf790* were AAA and GGA, respectively. A group II intron was identified in the mtDNA of *P. delicatissima*, located in the cytochrome c oxidase subunit genes (*cox1*). A putative gene *orf790* was found within the intron in the *cox1* gene. Comparative analysis of the mtDNAs of *P. delicatissima* and *P. multiseries* revealed that *P. multiseries* has 3 group II introns in the *cox1* gene (NC_027265) based on our re-annotation, with 3 ORFs (*orf714*, *orf742*, *orf790*) inserted in these introns of the *cox1* gene.

A Maximum Likelihood (ML) phylogenetic tree (Figure 1) was constructed using IQtree v1.6.12 (Jana et al. 2016) with 1000 bootstrap alignments based on tandem amino acid sequences of 31 common PCGs (including *atp6*, *8*, *9*; *cob*; *cox1*, *2*, *3*; *nad1*-*7*, *4L*, *9*, *11*; *rpl2*, *5*, *6*, *14*, *16*; *rps3*, *4*, *8*, *10*, *11*, *13*, *14*, *19*; and *tat*C) obtained from 36 publicly diatom mitochondrial genomes, including *Odontella regia* (MW018491), *Lithodesmium undulatum* (MW013551), and *Thalassiosira profunda* (MW013551) that we have recently constructed (Liu et al. 2021; Wang et al. 2021). *Phytophthora Ramorum* (EU427470) and *Saprolegnia Ferax* (NC_005984) were used as out-group taxa in the phylogenetic tree. As shown in Figure 1, all diatoms species were divided nicely into three clades corresponding to three classes of Coscinodiscophyceae, Mediophyceae, and Bacillariophyceae. As expected, *P. delicatissima* clustered well with *P. multiseries* with high support (100% bootstrap). Additionally*, P. delicatissima* and *P. multiseries* were clustered with *Fragilariopsis kerguelensis*, indicating a closer evolutionary relationship between *Pseudo-nitzschia* and *Fragilariopsis*. In conclusion, the complete mitochondrial genome of *P. delicatissima* could provide important information for understanding the phylogeny and evolution of diatoms.

**Figure 1.**
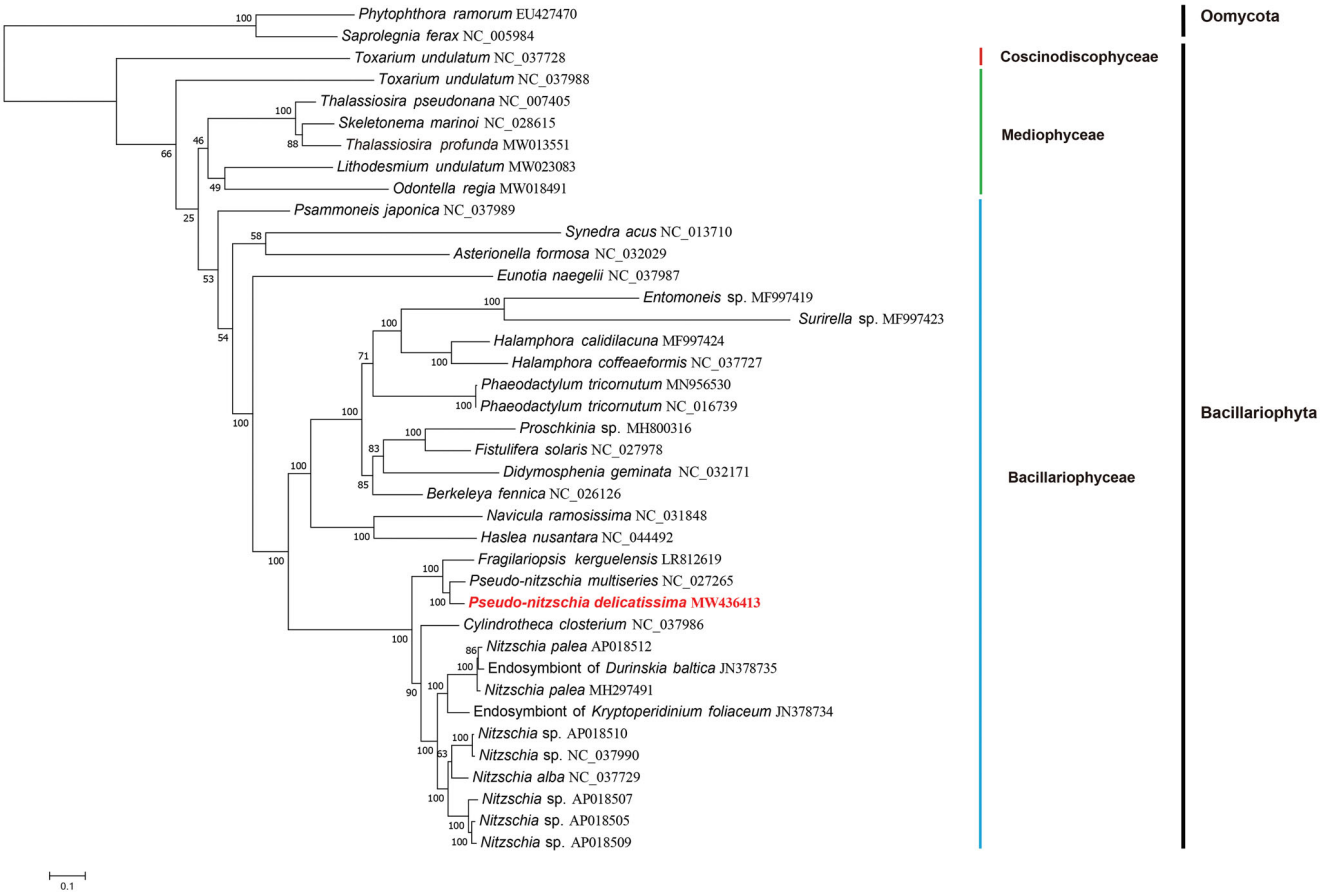
Maximum likelihood (ML) phylogenetic tree based on tandem amino acid sequences of 31 common PCGs from 36 diatom mitochondrial genomes, with *Phytophthora ramorum* (EU427470) and *Saprolegnia ferax* (NC_005984) were used as out-group taxa (bootstrap values based on 1000 replicates).

## Data Availability

The genome sequence data that support the findings of this study are openly available in GenBank of NCBI at (https://www.ncbi.nlm.nih.gov/) under the accession no. MW436413. The associated BioProject, SRA, and Bio-Sample numbers are PRJNA693874, SRR13500717, and SAMN17487522, respectively.
